# Supplementary material for: LRRK2 kinase mediates increased GCase activity in microglia in response to IFNγ-induced proinflammatory stimulation
Source: NPJ Parkinsons Dis. 2026 Mar 6;12:99. doi: 10.1038/s41531-026-01310-1 (PMC13087243; doi:10.1038/s41531-026-01310-1)
Supplement: Supplementary file 1 — Supplementary Information [file 41531_2026_1310_MOESM1_ESM.pdf]

LRRK2 Kinase Mediates Increased GCase Activity in Microglia in Response to IFNγ-induced Proinflammatory Stimulation

Emma J. MacDougall, Carol X.-Q. Chen, Eric Deneault, Zhipeng You, David Kalaydjian, Narges Abdian, Thomas M. Durcan, Konstantin Senkevich, Ziv Gan-Or, Edward A. Fon

Supplemental Figures

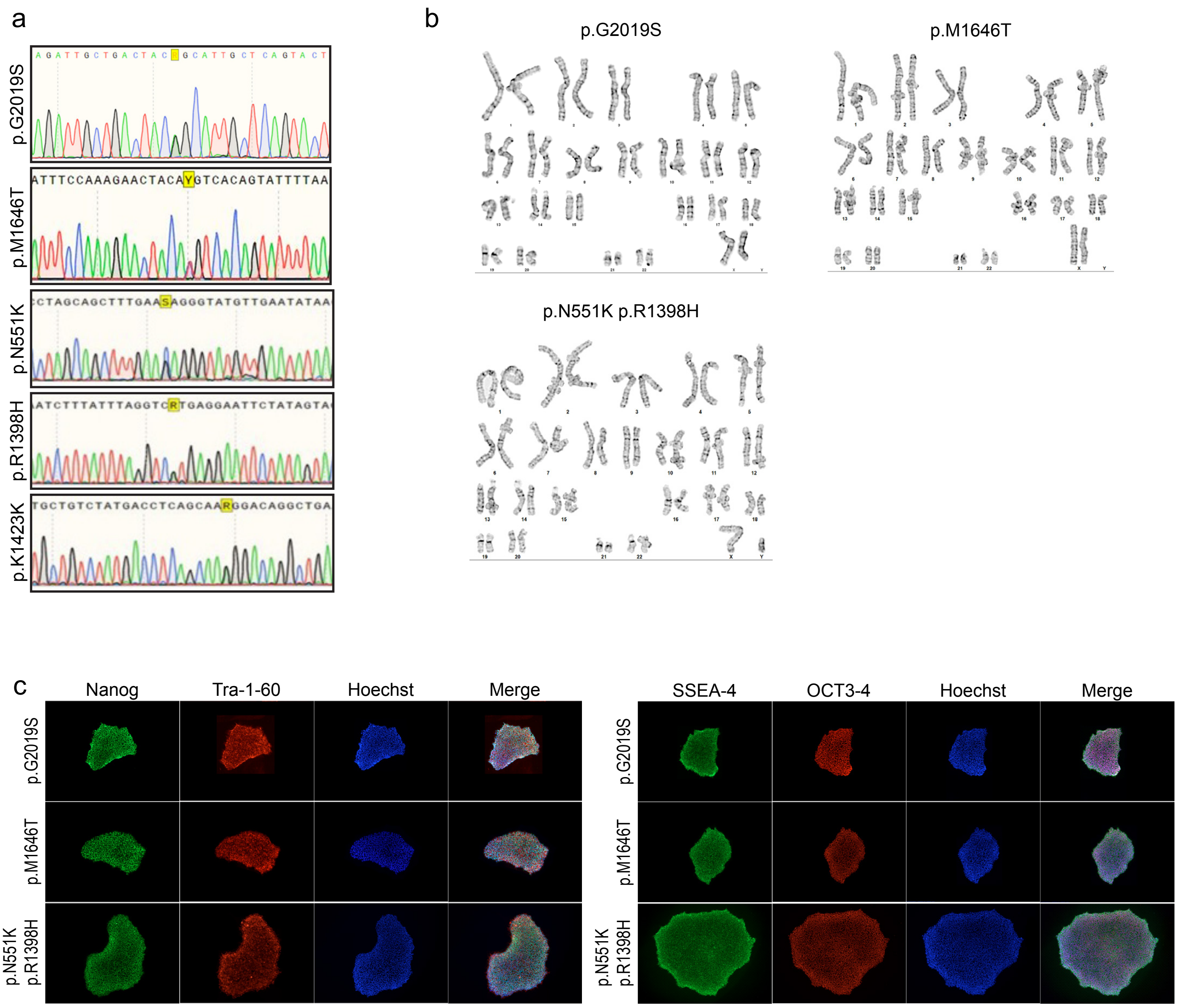

Figure S1 Sequencing and quality control of PD patient-derived LRRK2 variant iPSC lines used in this study. **a** Sanger sequencing confirmation of heterozygous LRRK2 variants. **b** Karyotype analysis shows no chromosomal abnormalities in iPSC lines. **c** Expression of pluripotency markers by IF staining.

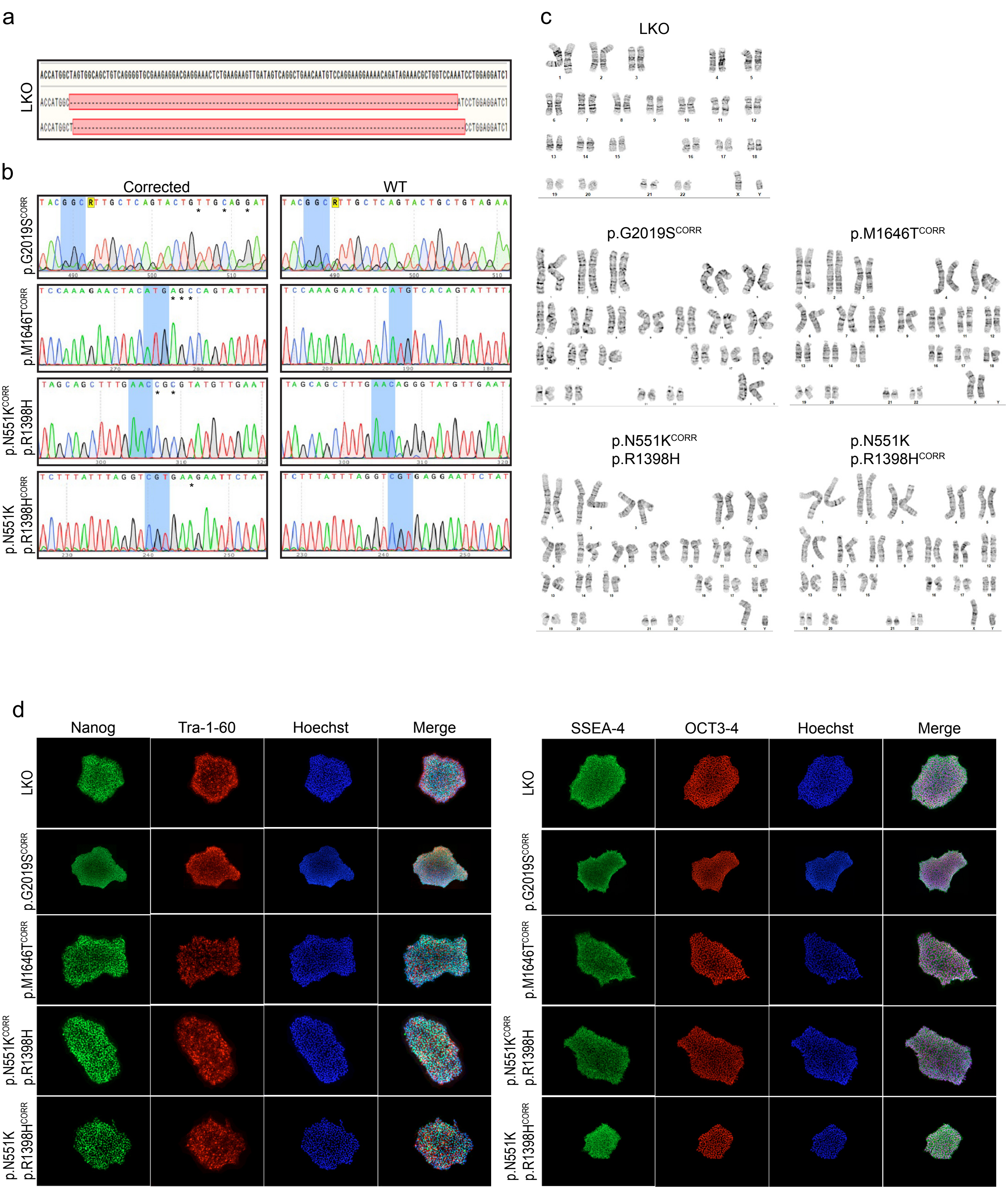

Figure S2 Sequencing and quality control of CRISPR-edited LKO and isogenic control iPSC lines used in this study. **a** Sanger sequencing confirms disruption of the LRRK2 gene by a 106 base-pair or 107 base-pair deletion, both resulting in frame shift mutations. **b** Sanger sequencing confirmation of correction of heterozygous LRRK2 variants, and introduction of PAM disrupting silent mutations. Corrected sequence illustrates the CRISPR-edited variant allele now corrected, with additional silent mutations indicated by *. WT sequence is that of the non-edited, non-variant allele. **c** Karyotype analysis shows no chromosomal abnormalities in iPSC lines. **d** Expression of pluripotency markers by IF staining.

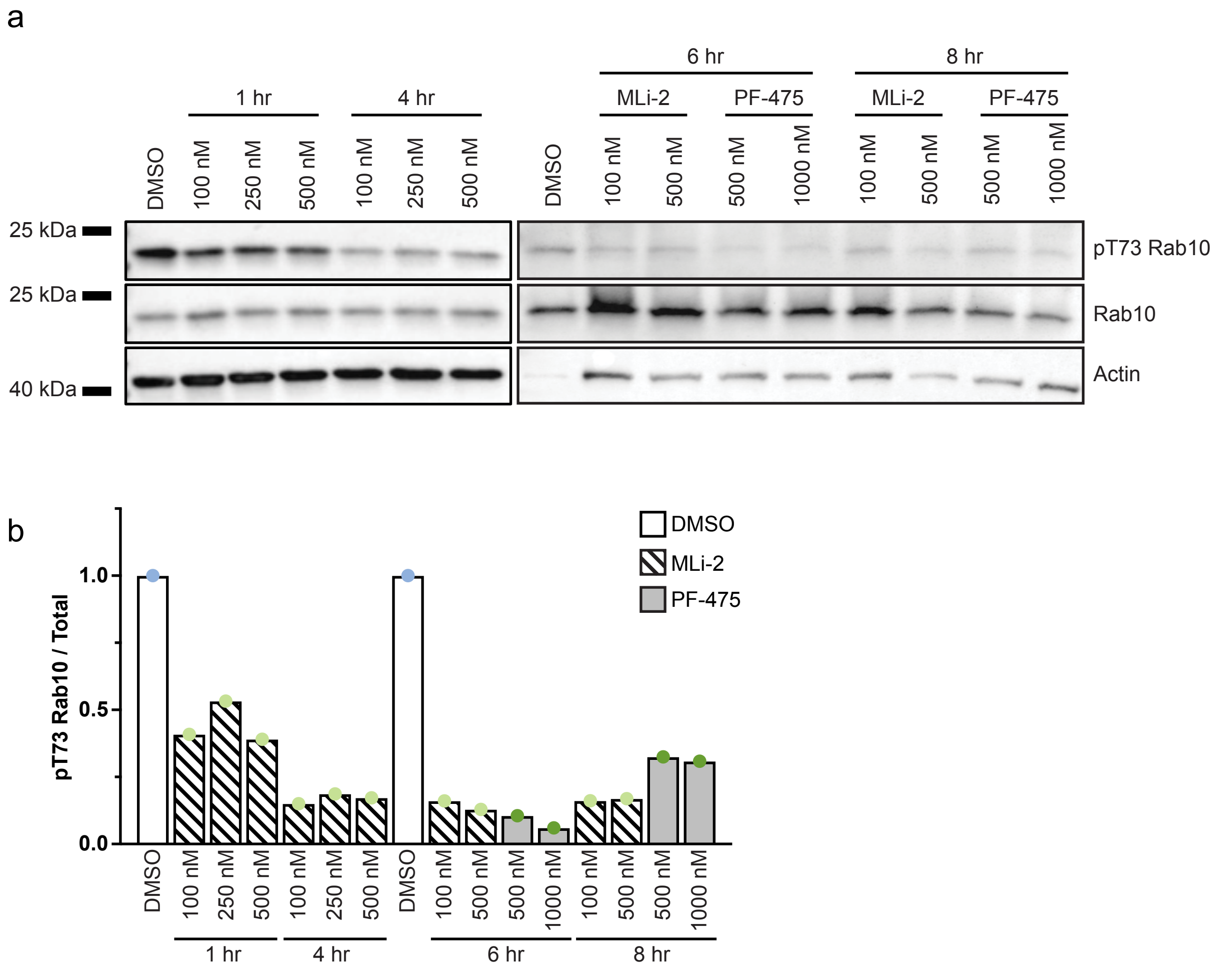

Figure S3 LRRK2 inhibition optimization. **a** Rab10 phosphorylation in LWT iMGs with or without MLi-2 or PF-475 treatment for 1, 4, 6, or 8 hr measured by WB. n =1 The membrane probed for pT73 Rab10 under 1 and 4 hour treatment conditions was stripped, cut, and reprobed for Rab10 and Actin. **b** Quantification of WB of phosphorylated Rab10 normalized to total Rab10.

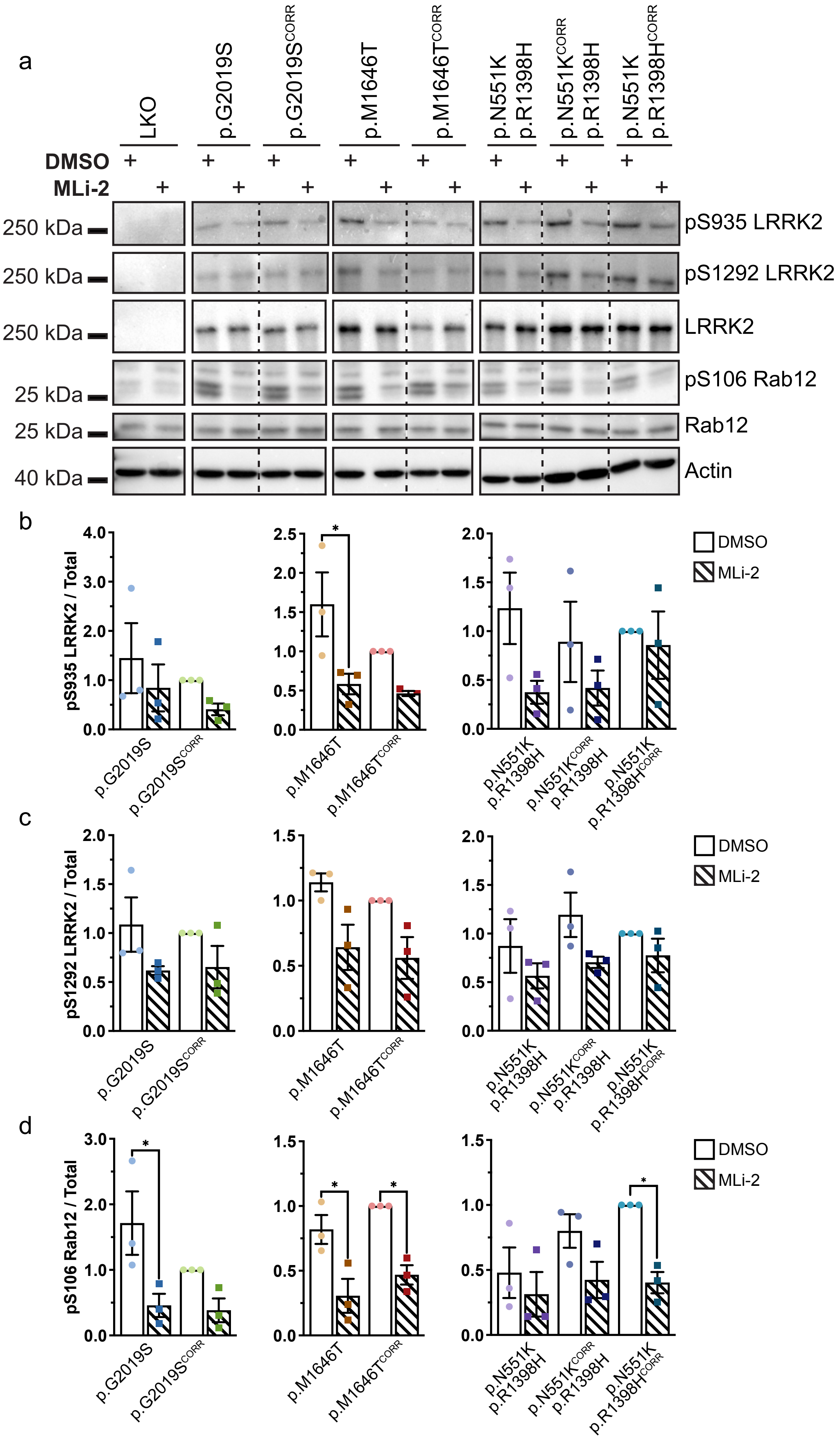

Figure S4 Rab12 and LRRK2 phosphorylation unchanged in LRRK2 variant iMGs. Expansion of data presented in Figure 2a **a** LRRK2 and Rab12 phosphorylation in LRRK2 variant iMGs with or without 6 hr 100 nM MLi-2 treatment as measured by WB. Membrane images have been cropped to remove additional inhibitor treatment and separate isogenic pairs. n = 3
b-d Quantification of WB of phosphorylated LRRK2 or Rab12 normalized to total LRRK2 or Rab12, levels normalized to isogenic control. **b** Quantification of pS1292 LRRK2. **c** Quantification of pS935 LRRK2. **d** Quantification of pS106 Rab12. One Way ANOVA with Bonferroni post-hoc test * $p < 0.05$, ** $p < 0.01$, **** $p < 0.0001$

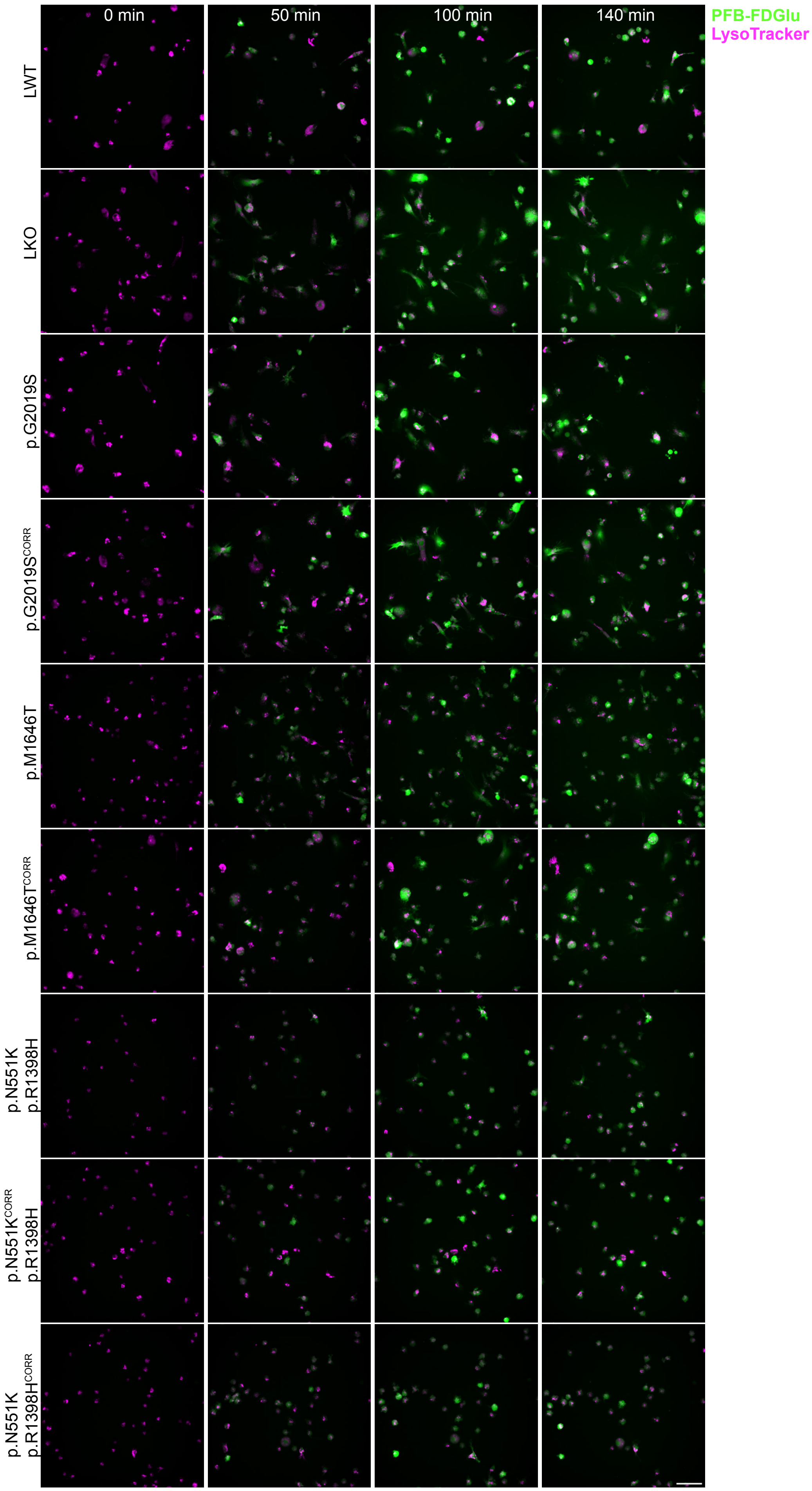

Figure S5 PFB-FDGlu GCase assay images from LRRK2 variant iMGs stained with lysotracker deep-red 0, 50, 100, and 140 minutes after dye-loading. Acquired using a 40X water immersion objective. Scale bar 50 µm.

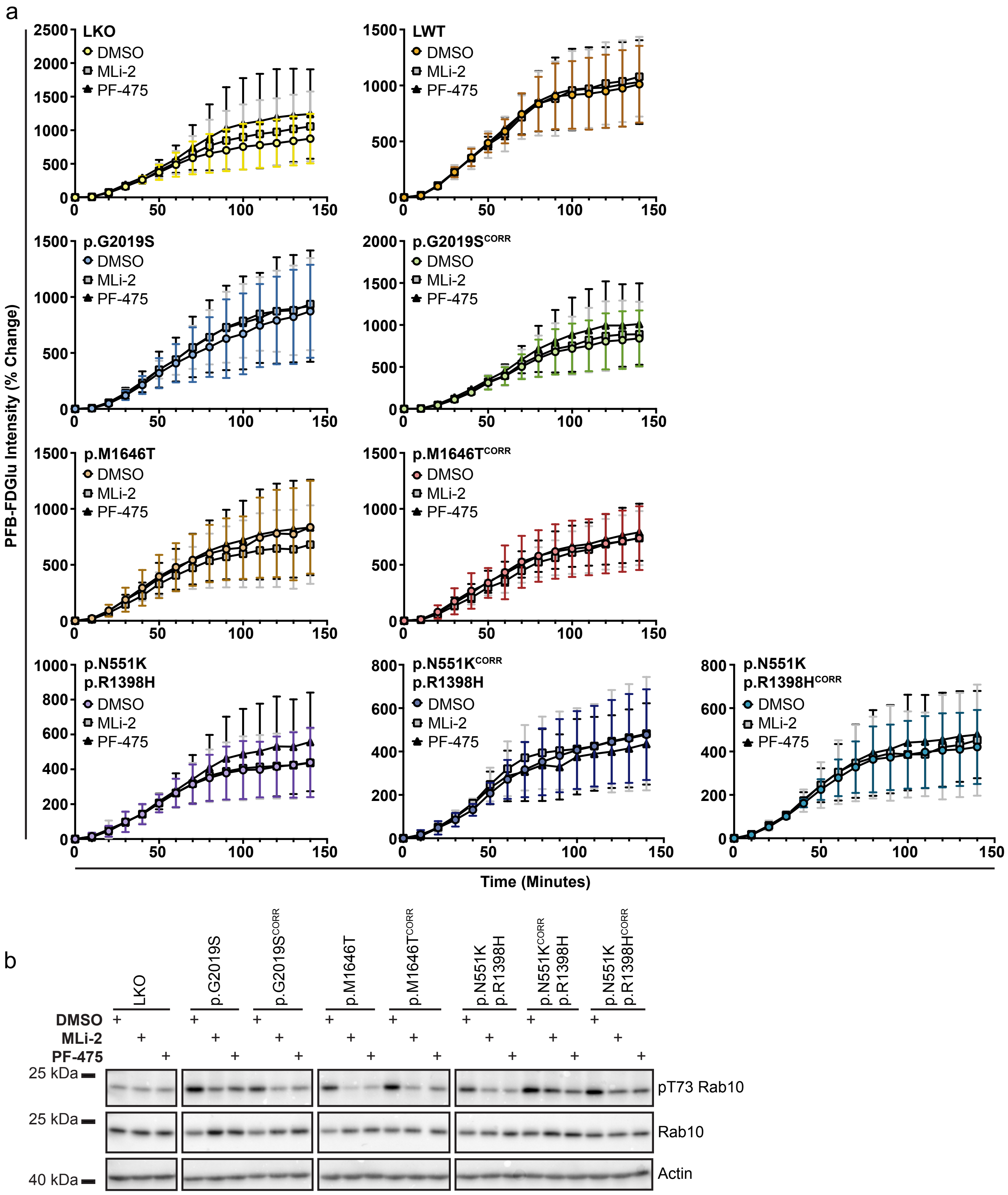

Figure S6 LRRK2 inhibition leads to Rab10 dephosphorylation but has no effect on GCase activity. **a** Percent change in mean PFB-FDGlu fluorescence per cell per well. n = 4. **b** Rab10 phosphorylation is decreased to a similar extent by 6 hr 100 nM MLi-2 or 500 nM PF-475 treatment. Membrane images have been cropped to separate isogenic pairs. n = 2 **a** Repeated Measures One Way ANOVA Tukey post-hoc test * $p < 0.05$, ** $p < 0.01$, **** $p < 0.0001$

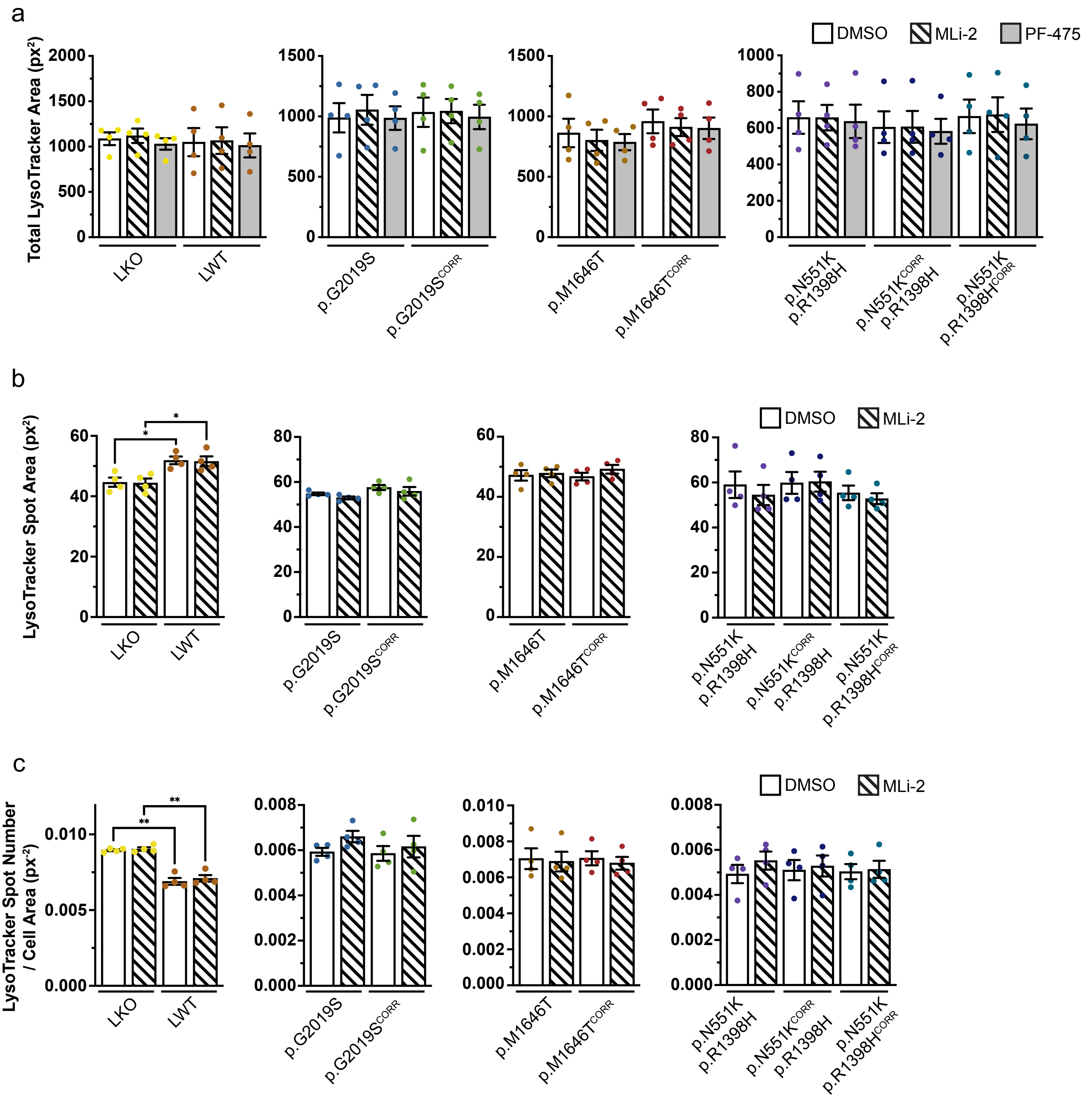

Figure S7 LRRK2 knockout, but not variants or inhibition, affect lysosomal mass. **a** Mean total lysotracker fluorescence area per cell per well (px2) at baseline (0 minutes). **b** Mean lysotracker spot area per cell per well (px2) at baseline (0 minutes). **c** Mean lysotracker spot number normalized to cell area per cell per well (px-2) at baseline (0 minutes). **a-c** Repeated Measures One Way ANOVA Tukey post-hoc test * $p < 0.05$, ** $p < 0.01$, **** $p < 0.0001$

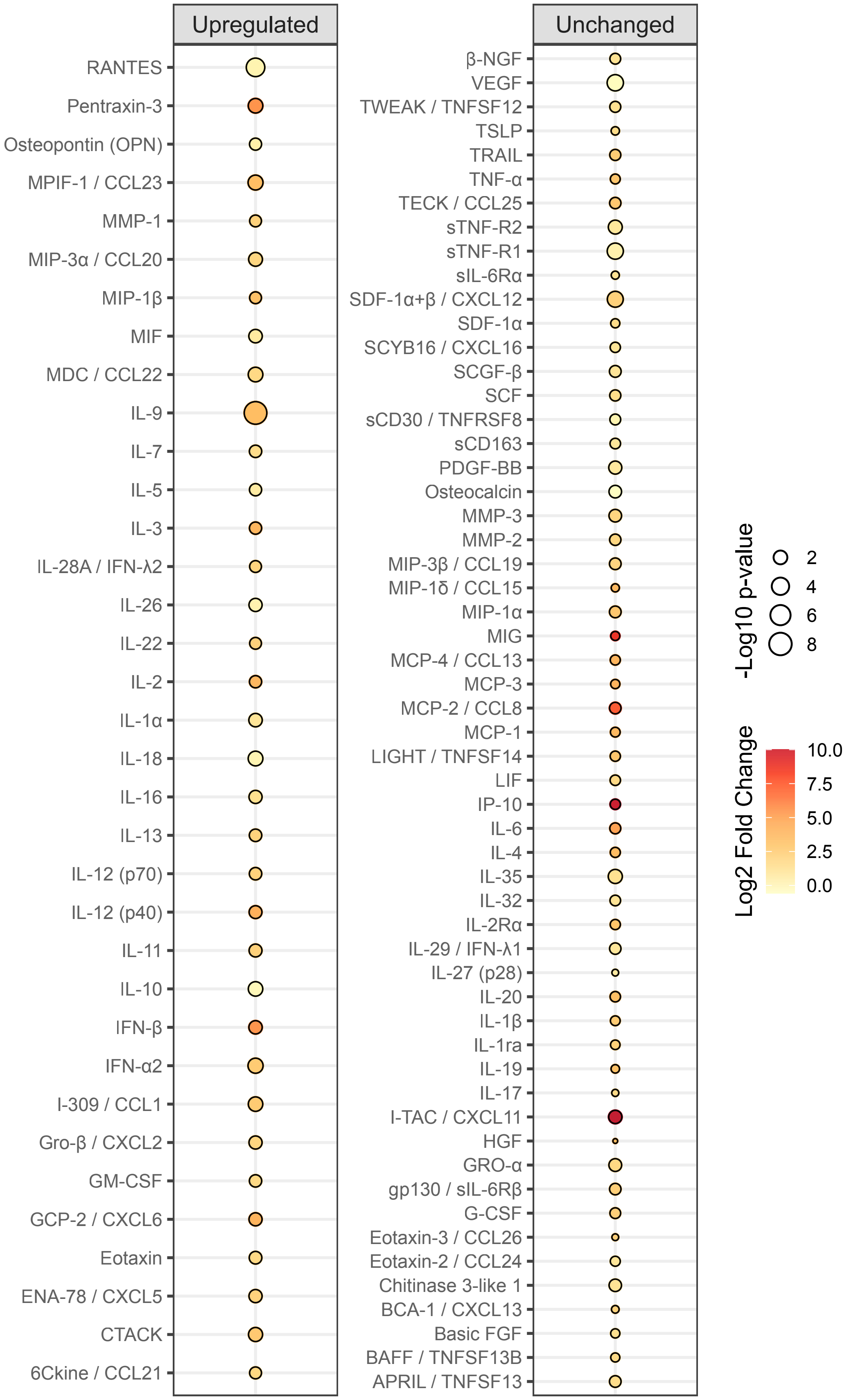

Figure S8 iMGs release proinflammatory cytokines in response to IFNγ treatment. Log2 fold change of concentration (pg/mL) of cytokines released by IFNγ treated LWT iMGs compared to vehicle treated LWT iMGs as measured by xMAP assay. n = 1. Log2 fold change cut-off > 1. Welch's t-test p-value cut-off < 0.05.

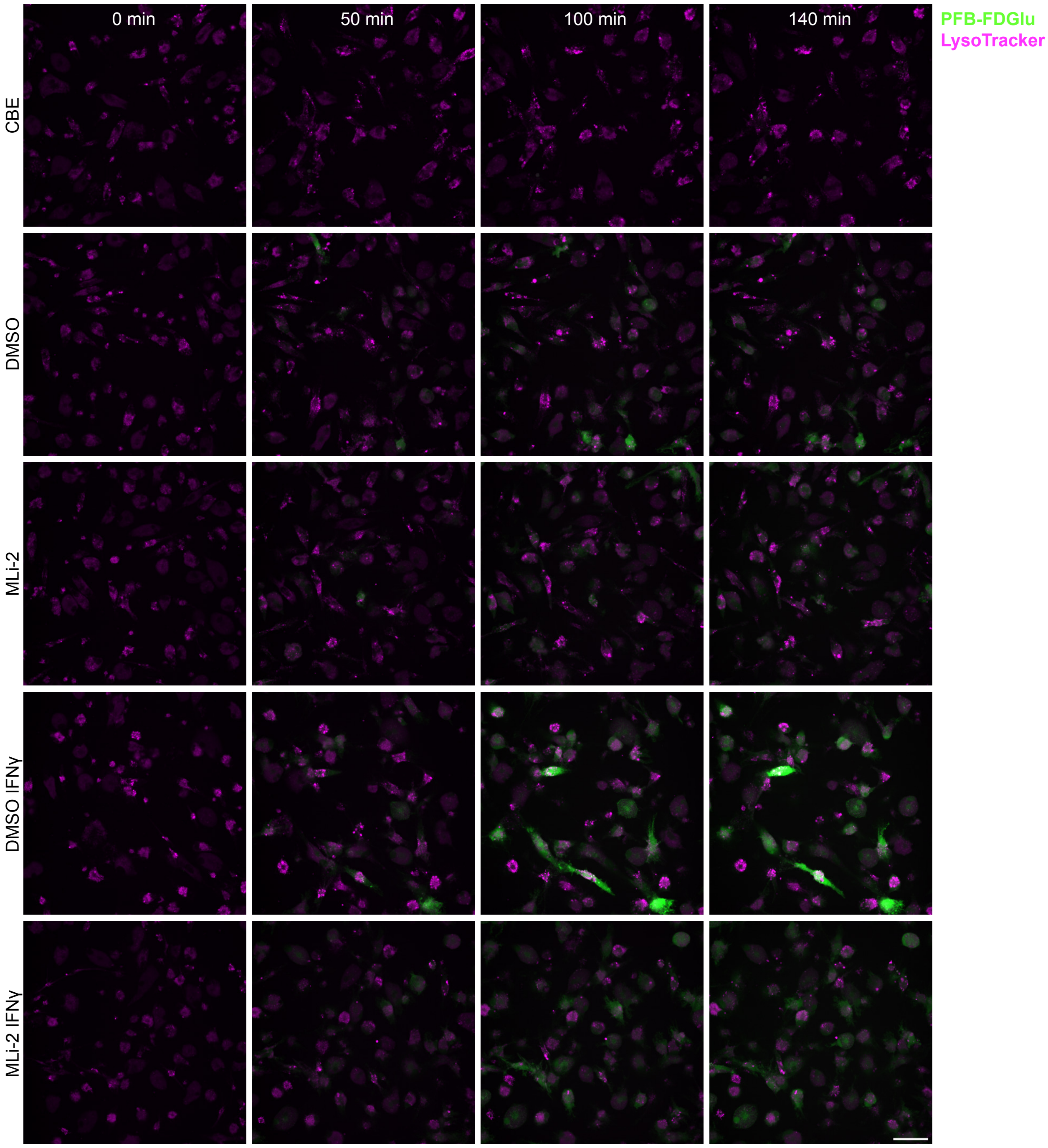

Figure S9 PFB-FDGlu GCase assay images from LWT iMGs treated with 20 ng/mL IFNγ and 100 nM MLi-2 stained with lysotracker deep-red 0, 50, 100, and 140 minutes after dye-loading. Acquired using a 40X water immersion objective. Scale bar 50 μm.

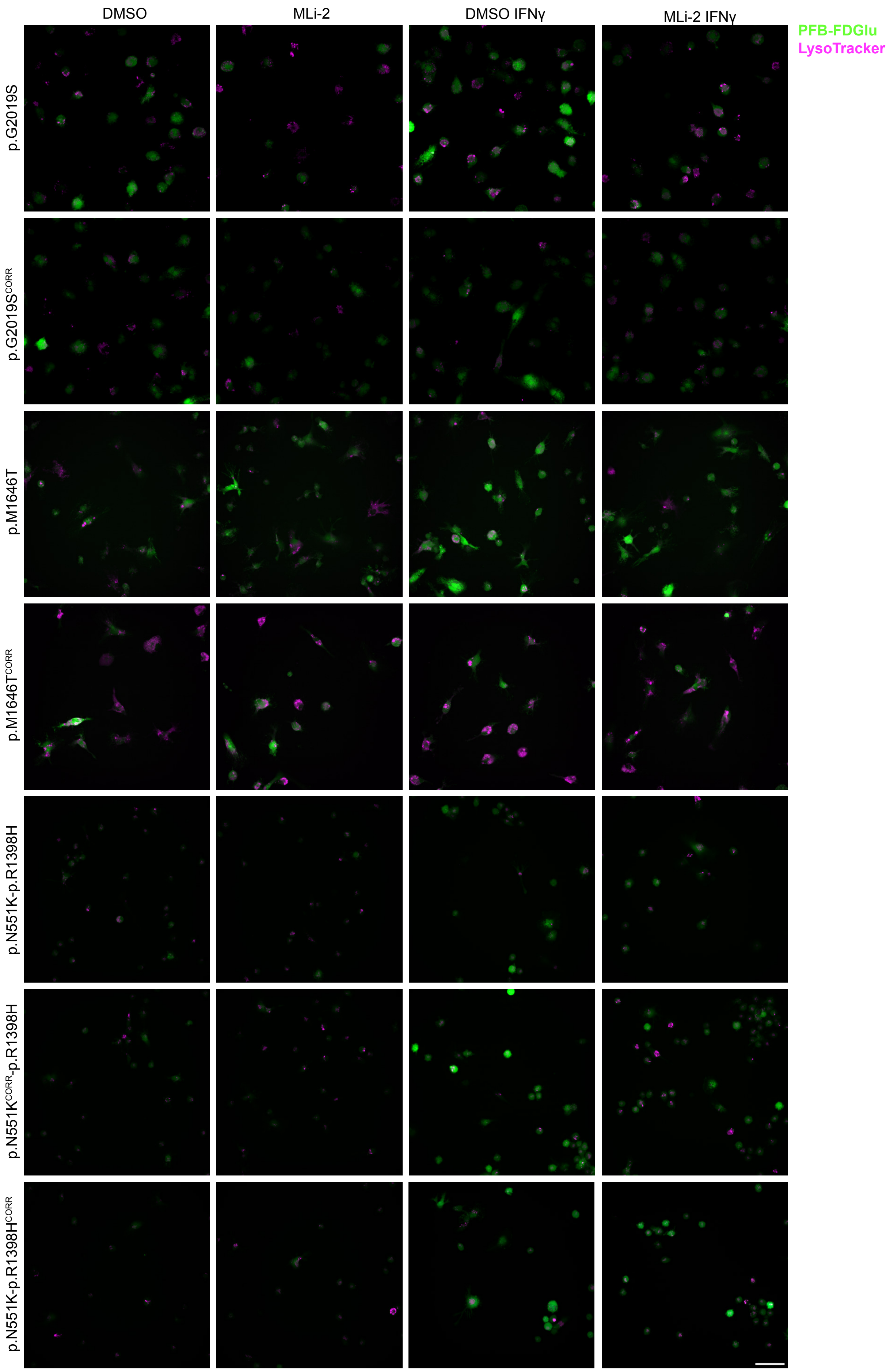

Figure S10 PFB-FDGlu GCase assay images from LRRK2 variant and isogenic control iMGs with or without 20 ng/mL IFNγ and/or 100 nM MLi-2, stained with lysotracker deep-red 140 minutes after dye-loading. Acquired using a 40X water immersion objective. Scale bar 50 µm.

LRRK2 Kinase Mediates Increased GCase Activity in Microglia in Response to IFNγ-induced Proinflammatory Stimulation

Emma J. MacDougall, Carol X.-Q. Chen, Eric Deneault, Zhipeng You, David Kalaydjian, Narges Abdian, Thomas M. Durcan, Konstantin Senkevich, Ziv Gan-Or, Edward A. Fon

Unprocessed Blot Images

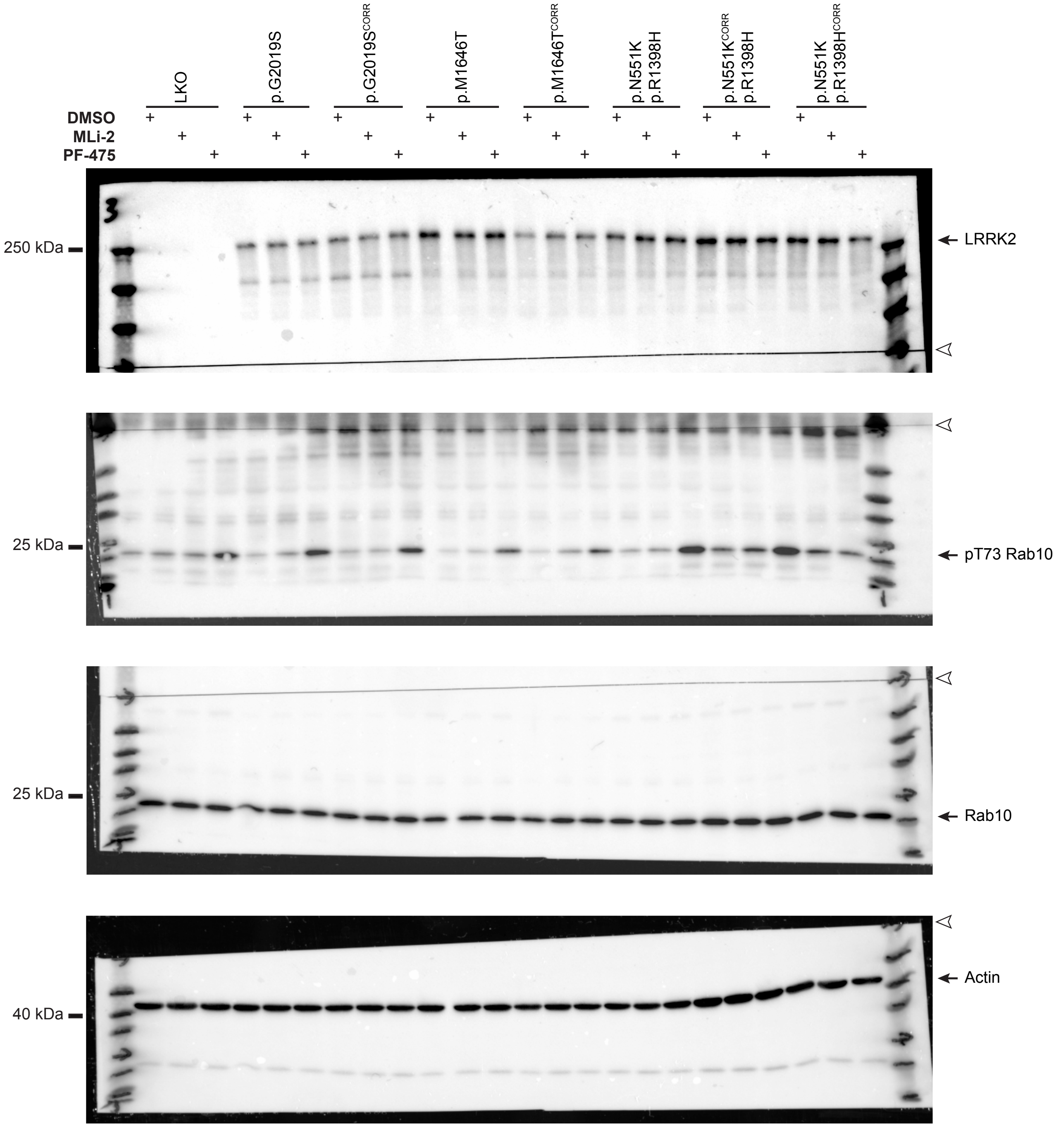

Unprocessed blot images from Figure 2a

Arrows indicate band of interest. Blots are cropped in the main figure to separate isogenic pairs and remove PF-475 treated samples. Open arrowheads indicate where membranes have been physcially cut to maximize the number of proteins assessed.

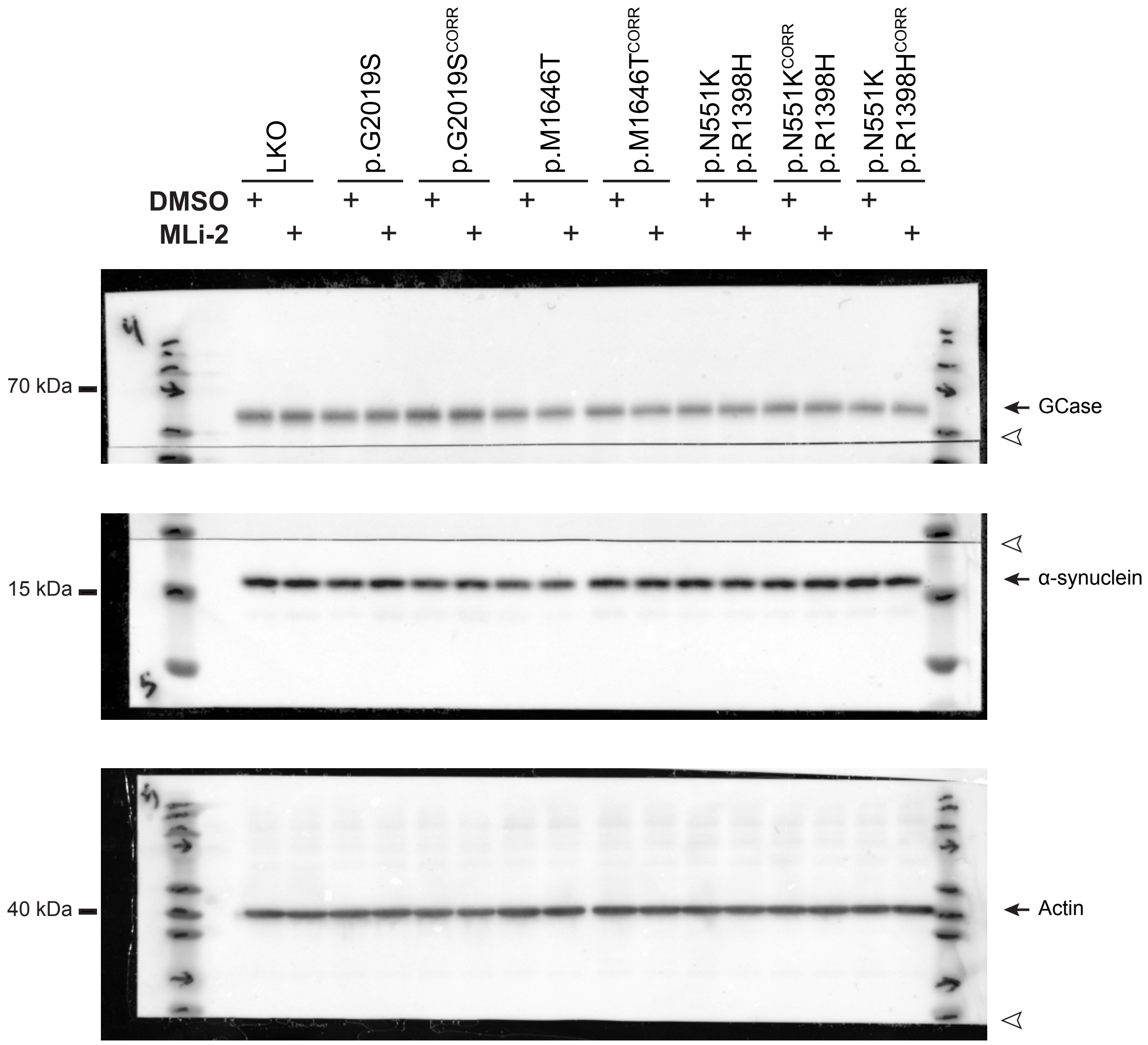

Unprocessed blot images from Figure 2c
Arrows indicate band of interest. Blots are cropped in the main figure to separate isogenic pairs. Open arrowheads indicate where membranes have been physcially cut to maximize the number of proteins assessed.

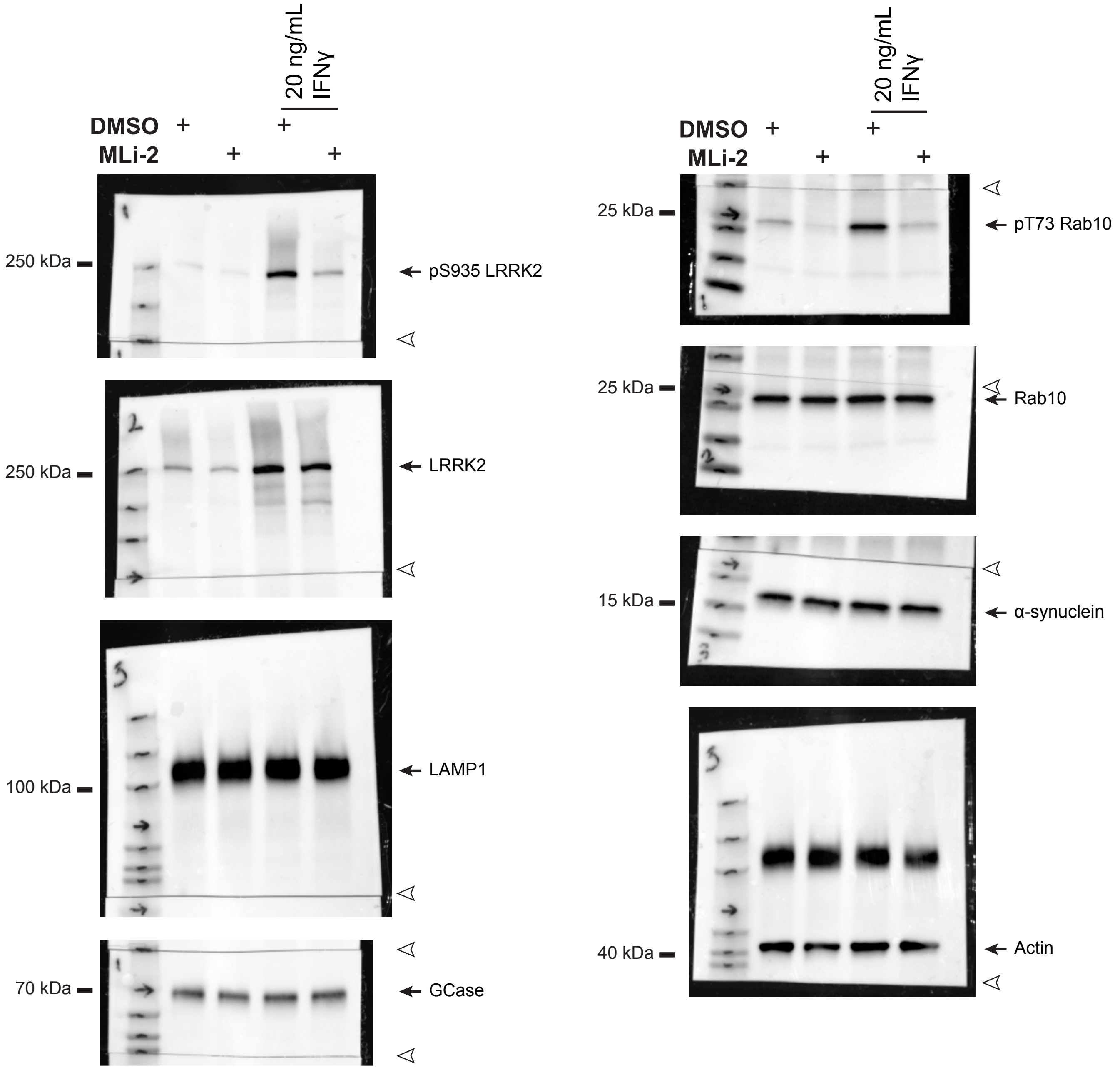

Unprocessed blot images from Figure 5a

Arrows indicate band of interest. Open arrowheads indicate where membranes have been physcially cut to maximize the number of proteins assessed. Membranes were reprobed with actin antibody.

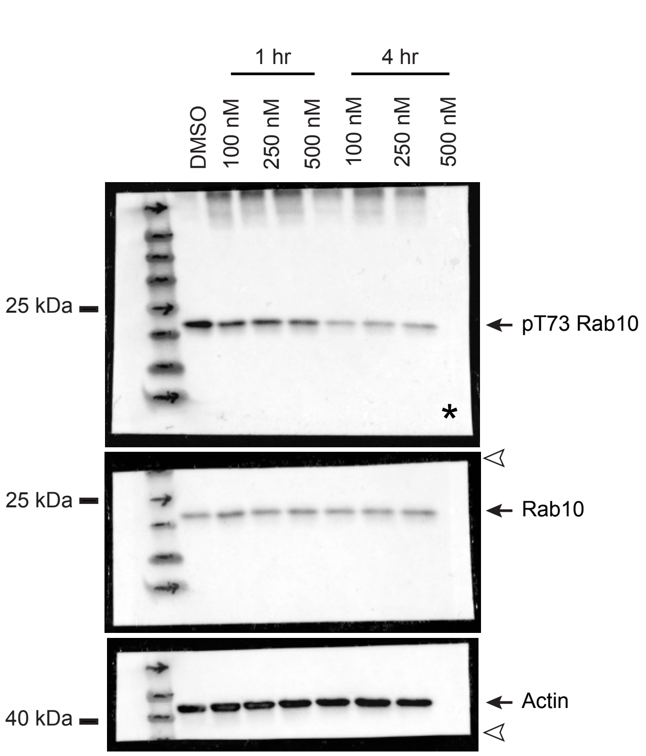

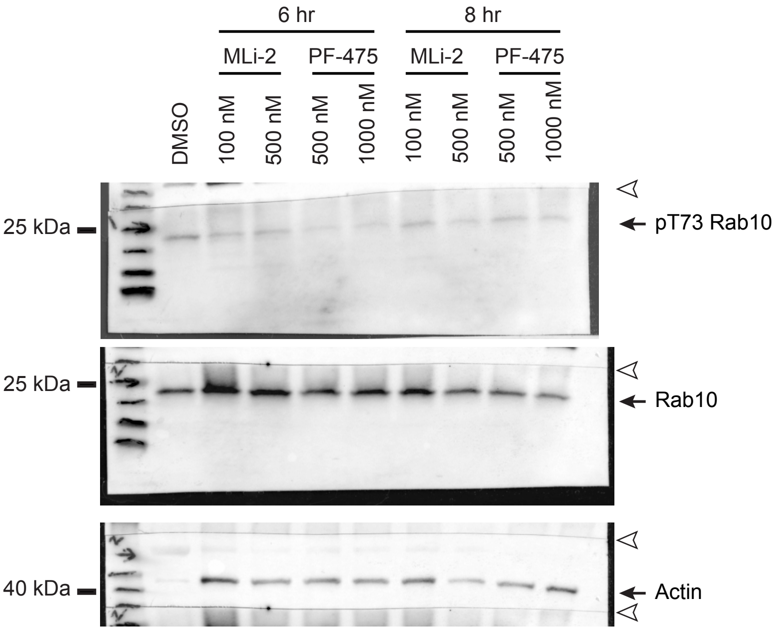

Unprocessed blot images from Supplementary Figure 3a

Arrows indicate band of interest. Open arrowheads indicate where membranes have been physcially cut to maximize the number of proteins assessed. The membrane indicated by * was stripped, cut and reprobed for total Rab10 and Actin.

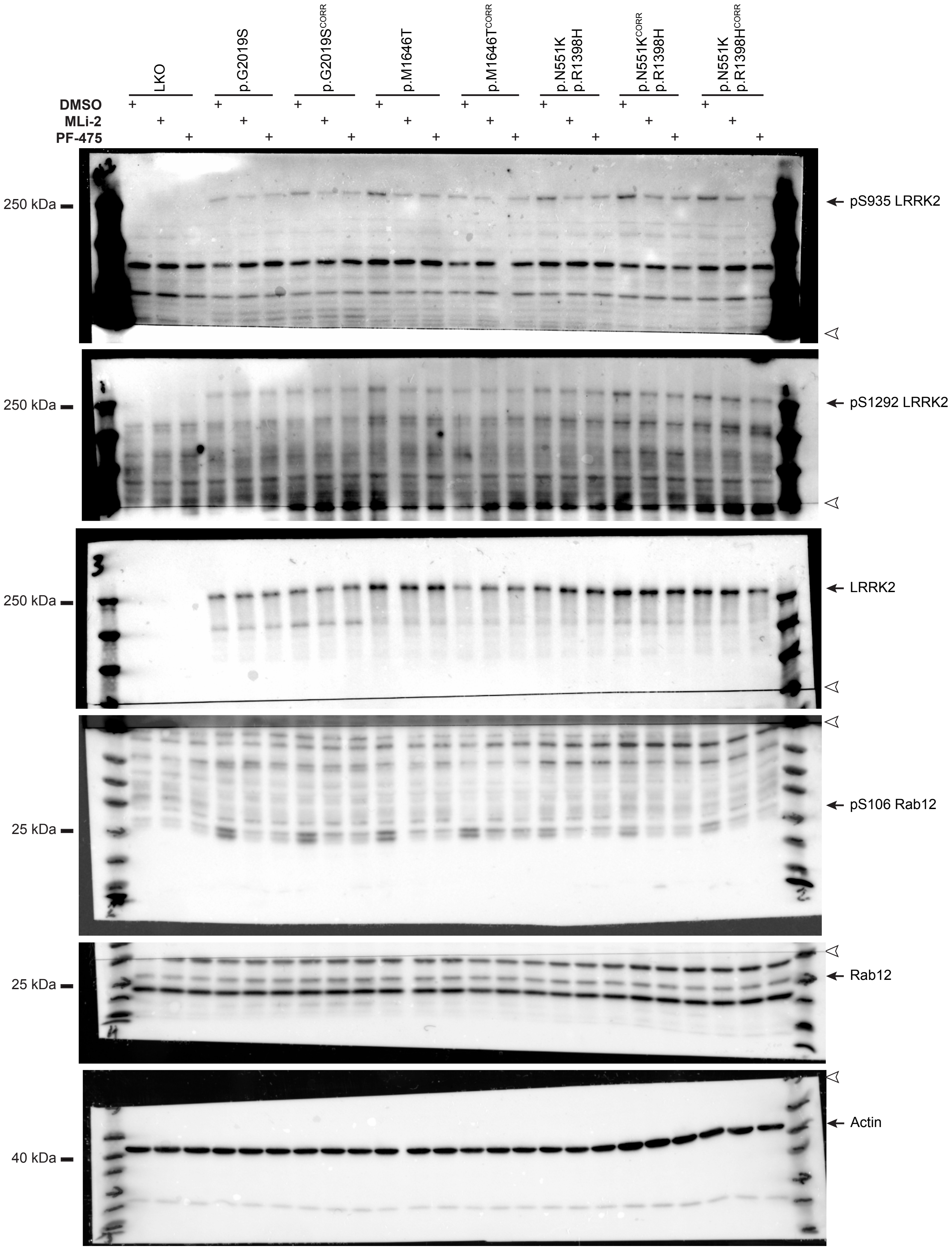

Unprocessed blot images from Supplemetal Figure 4a

Arrows indicate band of interest. Blots are cropped in the main figure to separate isogenic pairs and remove PF-475 treated samples. Open arrowheads indicate where membranes have been physcially cut to maximize the number of proteins assessed.

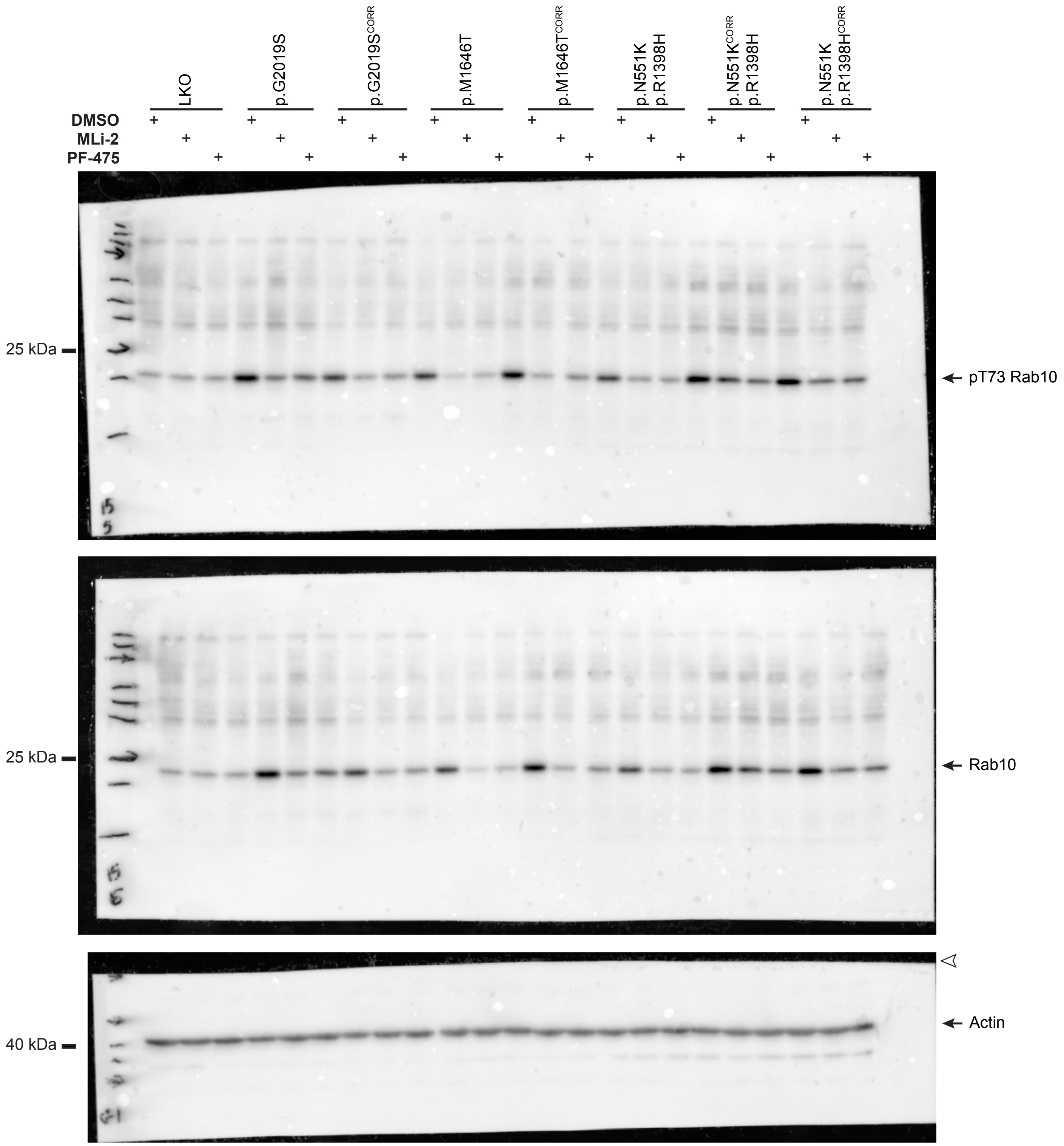

Unprocessed blot images from Supplemental Figure 6b
Arrows indicate band of interest. Blots are cropped in the main figure to separate isogenic pairs. Open arrowheads indicate where membranes have been physcially cut to maximize the number of proteins assessed.
